# AI-Assisted Detection of Biomarkers by Sensors and Biosensors for Early Diagnosis and Monitoring

**DOI:** 10.3390/bios14070356

**Published:** 2024-07-22

**Authors:** Tomasz Wasilewski, Wojciech Kamysz, Jacek Gębicki

**Affiliations:** 1 Department of Inorganic Chemistry, Faculty of Pharmacy, Medical University of Gdansk, Hallera 107, 80-416 Gdansk, Poland; 2 Department of Process Engineering and Chemical Technology, Faculty of Chemistry, Gdansk University of Technology, Narutowicza 11/12, 80-233 Gdansk, Poland; jacek.gebicki@pg.edu.pl

**Keywords:** sensors, biosensors, bioelectronics, biomarkers, artificial intelligence, machine learning

## Abstract

The steady progress in consumer electronics, together with improvement in microflow techniques, nanotechnology, and data processing, has led to implementation of cost-effective, user-friendly portable devices, which play the role of not only gadgets but also diagnostic tools. Moreover, numerous smart devices monitor patients’ health, and some of them are applied in point-of-care (PoC) tests as a reliable source of evaluation of a patient’s condition. Current diagnostic practices are still based on laboratory tests, preceded by the collection of biological samples, which are then tested in clinical conditions by trained personnel with specialistic equipment. In practice, collecting passive/active physiological and behavioral data from patients in real time and feeding them to artificial intelligence (AI) models can significantly improve the decision process regarding diagnosis and treatment procedures via the omission of conventional sampling and diagnostic procedures while also excluding the role of pathologists. A combination of conventional and novel methods of digital and traditional biomarker detection with portable, autonomous, and miniaturized devices can revolutionize medical diagnostics in the coming years. This article focuses on a comparison of traditional clinical practices with modern diagnostic techniques based on AI and machine learning (ML). The presented technologies will bypass laboratories and start being commercialized, which should lead to improvement or substitution of current diagnostic tools. Their application in PoC settings or as a consumer technology accessible to every patient appears to be a real possibility. Research in this field is expected to intensify in the coming years. Technological advancements in sensors and biosensors are anticipated to enable the continuous real-time analysis of various omics fields, fostering early disease detection and intervention strategies. The integration of AI with digital health platforms would enable predictive analysis and personalized healthcare, emphasizing the importance of interdisciplinary collaboration in related scientific fields.

## 1. Introduction

An indicator of a patient’s health condition and the base of diagnosis can be the presence and level of biomarkers, which are biological markers playing the role of a medical signature for a given patient. According to the dictionary of the U.S. Food and Drug Administration (FDA), in the document Biomarkers, EndpointS and other Tools (BEST), a biomarker is described as ‘a defined feature, which is measured as an indicator of natural biological processes, pathogenic or biological processes being a response to exposure or intervention, including therapeutic interventions’ [1]. The definition of digital markers is ambiguous and difficult to establish. It is suggested that the belief that they are an Internet extension of traditional biomarkers is a common mistake [2]. In practice, passively and digitally collected information does not have to be a statistical benchmark of health conditions but can be both a combination of data originating from the sensors and monitoring devices collecting numerical data and images that cover daily activities of the patients under different environmental conditions. For example, the results of measurement of the Transforming growth factor-beta (TGF-β) cytokine level as a biomarker of breast cancer should be considered as a traditional biomarker, regardless of the applied transducer employed for measurement and results collection [3]. On the other hand, digital evaluation of voice and breath quality falls within the category of digital biomarkers and is treated as multidimensional and time-varying information [4]. The differences between these biomarkers were presented recently by Babrak et al. [5]. The clinical interpretation of biological marker-related data is significantly influenced by such variables as sex, age, accompanying diseases, lifestyle, etc. Additionally, there is a significant difference in the way laboratories establish a threshold of biomarker concentration in order to classify patients and disease progress [6]. Traditional biomarkers continuously serve as valuable diagnostic and prognostic indicators for various diseases and health conditions. An increasing amount of research is exploring the diagnostic and prognostic capabilities of novel traditional biomarkers [7,8].

A high death rate caused by multifactorial diseases (cancers, respiratory tract diseases, cardiovascular disorders, mental disorders, and infectious diseases) results mainly from late diagnoses, which limits effective treatment and significantly increases the costs of healthcare [9,10]. The global healthcare market includes ambulatory and stationary care executed by clinicians, hospitals, and contractors, as well as self-care. Since the milestone of the 1950s, when Clark invented electrochemical glucose electrode, one can observe a substantial improvement in sensors for medical applications. Perfect examples are glucometers that do not require puncturing the skin and allow glucose levels to be monitored using a smartphone [11]. The adoption of electrochemical sensors, including biosensors and wearables, is anticipated to present new possibilities in medical diagnostics, wellness, and nutrition, which should support the transition from traditional diagnostic centers to decentralized, personalized, remote diagnostics. Personalized diagnostics is a domain where mobile devices can exert significant influence [12]. The rapid advancement of technology can be useful in disease diagnosis, but there is still a need for development that focuses on tools used for this purpose, such as sensors and biosensors for rapid and non-invasive diagnosis. However, despite a few decades of sensor development, their practical application in disease diagnostics still remains a challenge, since they require significant improvements to become precise diagnostic tools. This review study focuses on the integration of AI with portable, user-friendly devices, distinguishing it from previous surveys that addressed these aspects, namely AI [13,14] and biomarker detection [15,16,17], separately. As compared with recent reviews that cover similar topics [18,19], we explore how this combination can revolutionize diagnostic practices through continuous real-time analysis, enhancing diagnostic accuracy and reducing reliance on traditional laboratory settings. In this article, we have provided a comprehensive comparison of traditional and modern diagnostic techniques, highlighting the benefits of new technologies in terms of efficiency, accuracy, and accessibility. In addition, we have discussed the transition of these technologies from research to commercial applications, emphasizing their potential use in PoC settings and as consumer technologies. Finally, we offer a forward-looking perspective on advancements in sensor technology and biosensors, predicting their impact on early disease detection and personalized healthcare.

**Figure 1:**
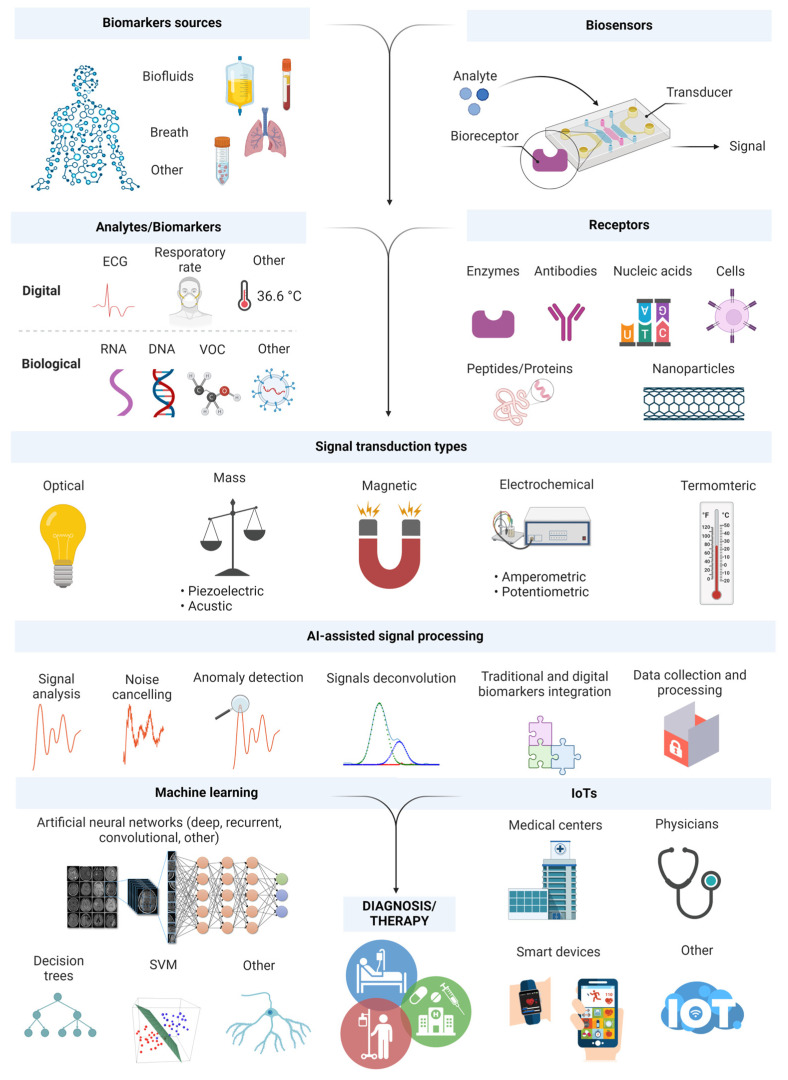
A schematic representation of (bio)sensor components for detecting biomarkers. ML- and AI-based data processing enables integration and combination of traditional biomarkers with digital ones to personalize healthcare. The acquired data can then be collected, distributed, and evaluated by clinicians and individual patients. Created with BioRender.com.

Progress in sensor technology should be accompanied by the adoption of a more holistic approach to data collection and analysis. A rapid increase in the popularity of smart devices and wearables can facilitate monitoring of biomarker level; hence, it is necessary to elaborate unified, user-friendly tools, which pave the way for clinical implementations. Currently, more than ever, it is important to deepen the knowledge of the correlation between different biomarkers and particular diseases via AI and advanced ML. Figure 1 illustrates the operation and structure of typical (bio)sensors where data processing can be AI-assisted and data integration is achieved using the Internet of Things (IoTs), allowing collection, processing, and exchange of data without human intervention. Moreover, taking into account polymic (metabolomic, proteomic, genomic, and transcriptomic) patient signatures coupled with pathological and clinical data will help to determine the optimum level of key biomarkers. Consequently, it should contribute to the training of future sensor technologies based on AI and/or design of the multicomponent (bio)sensors for the generation of clinically significant and personalized output data. This would provide information beyond the knowledge of clinical experts and pathologists. It is necessary to provide a critical perspective of AI applications to improve diagnostics, employing a holistic approach, which can channel the research on (bio)sensors towards their clinical application in the near future.

## 2. Challenges in Translational Medicine, Bringing Biomedical Science into Clinical Practice

**Figure 2:**
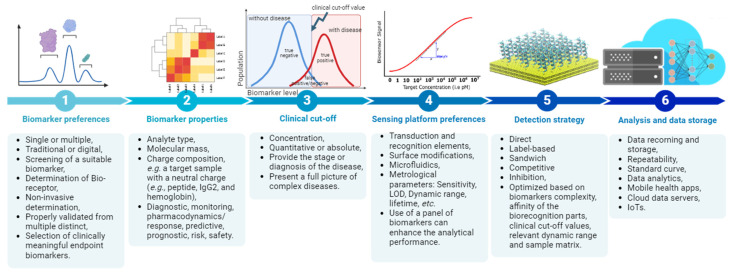
The key stages during the development of diagnostic tools based on sensors and biosensors.

Translational medicine deals with transferring scientific research results to practical application in disease diagnostics, treatment, and prevention. It aims at increasing the effectiveness of therapy and improvement of patients’ conditions via acceleration of the transfer of modern medical technologies from research laboratories to clinical practice. The European Society for Translational Medicine (EUSTM) defined three keystones of translational medicine: (i) scientists, (ii) clinicians, and (iii) community, including non-profit foundations, universities, and corporations providing medical equipment/services and medicines [20]. The main goal is to discover and test innovative pharmacotherapies, devices, and treatment methods. However, our understanding of some diseases is limited to specific biomarkers, thus hindering the transfer of laboratory results to clinical practice [21]. Moreover, a bottleneck is a gap between required knowledge/technologies and discoveries/inventions, namely a correlation between the needs defined by clinicians, governments, organizations, and research activities undertaken by scientists during development investigations. Implementation of scientific research results in clinical practice generates high costs. There is a lack of regulatory supervision and public support for their dissemination, as well as a shortage of reliable data from open sources, which significantly impairs clinical transfer of biomedical research. The complications of reporting research directions aimed at transfer between laboratories and clinical practice, the so-called bench-to-bedside ones, are overwhelming [18]. For example, diagnostic tests or other medical procedures, conducted at one site—usually to improve comfort or reduce cost—restrict acquisition of prognostic data on traditional biomarkers, especially from patients in a terminal state. Moreover, their validation and utilization due to digital results of biomarker investigation is difficult to execute and costly. It should also be emphasized that medium and small private medical facilities usually do not find justification for cost and economic risk associated with clinical transfer, while recognized corporations and institutions financed by the government and capable of generating implementation-worthy results prefer close access to such data. The increasing popularity of health applications for smartphones and smart devices constitutes a new source of data acquisition and processing as far as digital biomarkers are concerned. The widespread use of health applications via smartphones (e.g., mHealth) has emerged, driven partly by the COVID-19 pandemic, as well as by concerns regarding data privacy risks. Iwaya et al. [22] studied 27 top-ranked mental health apps, underscoring significant privacy vulnerabilities and urging developers to prioritize privacy considerations and users to demand privacy-friendly app solutions. Also, insufficient data and knowledge networks for exchange between engaged companies, including industry, academic communities, doctors, patients, regulatory agencies, and technological companies, increase the complexity of the real transfer of biomedical research to clinical practice [5]. Moreover, the lack of clarity on digital biomarker classification, the diversity of social groups, and their interaction with environmental factors as well as the lack of connection with traditional biomarkers significantly restrict clinical transfer of biomedical devices, which are treated as ‘pseudo-biomedical’ ones that do not guarantee reliable data. There is also an urgent need for identification of differences between traditional and digital biomarkers as well as a need for system evaluation of how they can be employed either individually or after a suitable combination to describe the health state of the patients. The gradually evolving field of digital biomarkers converges with traditional biomarkers in biomedical applications, for instance, in evaluation of the early stages of Alzheimer’s disease [23,24,25]. Clinical and research communities would certainly benefit from more interest in these technologies. However, the key factor is understanding of the fundamentals of their operation, limitations, and applicability in the newest bioelectronic diagnostic devices supported by AI/ML as the modern tools in clinical diagnostics [18,26]. Before implementation in clinical practice, the biosensors must go through many complex tests aimed at confirming their reliability, safety, and other parameters. There are many stages of clinical evaluation of the biosensors (Figure 2).

For the detection of biomarkers present at trace concentrations, microfluidic and multicomponent devices comprising multiple stages and extended reaction times are needed. This approach streamlines label-less detection techniques and holds considerable promise in healthcare applications [27,28]. Recently, a huge demand has been seen for miniaturized microfluidic sensing platforms for portable PoC diagnostics, achieved by integrating lab-on-a-chip technology and electrochemical analysis. However, developing small, integrated, and reliable sensors capable of conducting multiple and simultaneous electrochemical analyses in a single device remains a challenge. Simultaneous microfluidic electrochemical biosensing systems designed to detect multiple biomarkers within a single device are in demand [29]. A design by Lee et al. facilitates loading of multiple reagents for simultaneous analyses. Using a similar microfluidic electrochemical sensor system, it is possible to successfully identify multiple biomarkers. Such innovative approaches provide new platforms for rapid, miniaturized, and sensitive diagnostic sensing within a single device for various human diseases [29,30,31]. Design and working principles of microfluidic sensing platforms for biomarker detection were recently presented by Mitchell et al. [16].

The sequential approach to biomarker selection offers flexibility in choosing platforms and detection methods for assay developers. High-throughput sample analysis is crucial for studying panel biomarkers, but challenges arise when biomarkers in the panel have either diverse structures or physical properties. Adjustments in dynamic range may be necessary to accommodate different biomarker types. Advanced signal processing and integration with ML algorithms are expected to enhance detection accuracy in high-throughput biosensors for panel biomarker analysis in the future [32]. One of the first is the so-called proof of concept, preliminary investigation aimed at confirming that a new concept has application potential [33]. It allows initial evaluation of effectiveness and safety. This stage typically involves the design and evaluation of biosensor operation in laboratory conditions. In order to ensure that the biosensor can reliably and precisely identify target biomarkers, it is tested on selected biological samples. After this stage, follow preclinical tests on animal models, clinical validation verifies diagnostic usefulness on selected groups of patients in clinical conditions. A comparison with routine techniques has been used to evaluate this issue. Now that the safety and effectiveness have been demonstrated in clinical tests, the biosensor must be submitted for acceptance by the regulatory authorities, before it can be admitted to the market and commercialized. To ensure the safety and effectiveness of biosensors in clinical practice, their evaluation is a complex and multistage procedure involving the cooperation between scientists, clinicians, and regulatory authorities. Available databases of clinical research currently inform more than 20 biosensors during clinical evaluation, which operate independently or in connection with AI/ML to trace the traditional and digital biomarkers https://clinicaltrials.gov (accessed on 8 May 2024) [26].

### Criteria for Sensing Platforms in Translational Medicine

The World Health Organization (WHO) introduced the criteria aimed at increasing the effectiveness of early diagnostics of diseases, which include affordability, sensitivity, specificity, user-friendliness, rapidity, reliability, and availability as the fundamental features for evaluation of the diagnostic tests [34]. Other requirements for the devices operating in the PoC mode are small dimensions, the possibility of multiplex analysis, multiple uses, and configuration for continuous monitoring (for example, wearable devices). The aforementioned points of emphasis influence the development of the sensor systems, which seem to be the most suitable tools due to PoC operation, rapidity, specificity, sensitivity, possibility of miniaturization, affordability, etc. [35]. Using sensors as analytical devices, it is possible to detect the target compounds (ligands—biomarkers) via conversion of molecular recognition into measurable, easily interpretable analytical signals [36]. They are composed of two basic elements—a primary transducer that acts as a biological receptor element, and a secondary transducer that converts the response of the primary transducer. [37]. The type and specification of the transducer can differ depending on the kind of biomarker and target application. For instance, wearable devices may require more flexible and durable materials as well as wireless transmission in real time with automatic data processing; PoC diagnostic tests may compromise certain aspects, such as flexibility and wireless data transmission [38]. The acceleration of the seamless transfer of biosensors from the laboratory to real-world applications requires the evaluation of their metrological parameters, such as limit of detection/quantification, durability, sensitivity, specificity, and more. Mobility of the detection systems is one of the main factors hindering clinical transition and commercialization, as well as computing power, wireless transmission, and availability of a suitable sample or patient preparation. The lifetime of a device and the reagents is also a challenge.

Devices with their own power source have gained increasing popularity, although their stability and durability are still far from optimum. Recent research resulted in the elaboration of AI-based nanogenerators, to overcome limitations connected with the analysis, design, and production of piezoelectric and triboelectric nanogenerators [39]. It is predicted that such devices based on AI/ML would exhibit increased mechanical and electrical efficiency, helpful in elaboration of wearable biosensors with their own power source. BioScreen is a recent example of a fully portable pathogen biosensor with its own power source operating in the PoC mode [40]. The cost of the sensing platform can vary depending on a few factors, including the complexity of the device, materials used for the construction of a transducer, and recognition elements applied. Now and then, these factors can increase the cost of biosensors. Similarly to all new products, biosensors can become cheaper with progress in technology and with increasing demand. Then, they will be more affordable and more practical over a wider application range. With clinical applications, validation of the laboratory results on a bigger target group, namely on a bigger number of samples/patients, is indispensable for standardization and commercialization [41,42]. The IoTs represents a significant advancement in remote medical monitoring, with wearable biomarker sensors having been developed to enable both PoC diagnosis and continuous disease management. These sensors offer dynamic sampling and analysis of biomarkers in biofluids, providing high sensitivity, flexibility, and cost-effectiveness. Data from these sensors can be transmitted to a smartphone or laptop and then to the cloud for storage, processing, and retrieval before being displayed on customary applications. Wearable IoT biomarker sensors are particularly valuable for early disease diagnosis and continuous monitoring in regions with limited access to healthcare [42]. With the rise in information and communication technology, the concept of an Internet of Medical Things (IoMT) has attracted growing attention over recent years. The significance of routine physiological metric monitoring and intelligent data analysis for the early detection and prevention of diseases has been emphasized [43]. Printed electronics enabled the development of flexible devices and wireless body sensor networks capable of continuously gathering various physiological data. Monitoring diverse physiological signals requires multiple biosensors and devices that increase equipment costs and user inconvenience. Thus, there is a need to design a single device with multiple sensor combinations for continuous physiological signal monitoring. Advancements in IoMT, together with precision medicine, have the potential to revolutionize healthcare, especially in terms of regular physiological monitoring and risk evaluation. The use of IoMT and wearable electronics is anticipated to mitigate challenges faced by the conventional healthcare system, including staff shortages and high medical costs. IoMT holds promise as a technology capable of enhancing overall health, potentially extending human lifespan, and averting chronic illnesses.

Main metrological parameters of sensors (e.g., LOD, sensitivity, lifetime, etc.) are closely related to devices for the detection and monitoring of traditional biomarkers, but their evaluation must also be implemented in digital biomarker applications. Improvements in nanotechnology and flexible electronics together with the progress in supplementary technologies, such as microflows and wireless data transfer, can enhance the characteristics of transducers with a simultaneous significant decrease in their cost. Appropriate transducers, serving as critical components in biosensor development, convert biological signals into detectable outputs. In addition to appropriate bioreceptors and miniaturized readout electronics, transducers significantly affect the functionality and design of wearable devices for personal health monitoring. The transducers in modern biosensors should be compatible with biological and electronic elements as well as with non-conventional substrates, such as leather or fabrics [44]. They are also expected to monitor traditional and digital biomarkers in a continuous and non-invasive mode, without deterioration of sensitivity and specificity. Moreover, in the future, wearable devices will be able to integrate two different transducers for simultaneous detection of traditional and digital biomarkers. For example, traditional biomarkers, occurring at trace concentrations, require highly sensitive transducers, such as capacitance or optical ones, while digital biomarkers, including acoustic, vibration, or thermal ones, can employ piezoelectric or calorimetric transducers. Cooperation between such transducers enables a versatile approach to a particular disease. As compared to conventional tests, they provide a cheap and simplified alternative to time-consuming laboratory analyses. Their successful implementation depends on the development of biotechnology, micro-/nanotechnology and microelectronics, supramolecular chemistry, computation and chemometric techniques, etc., which determine the improvement of sensors’ metrological parameters, so they can be more useful in practical diagnostics of diseases [45] and more environmentally friendly [46]. Clinical application of an innovative generation of (bio)sensors requires evaluation of their clinical accuracy (i.e., comparison with the results of standard clinical procedures) and analytical accuracy (i.e., comparison of differences against the reference results obtained with recognized techniques).

While many smart devices effectively monitor digital biomarkers, only a few extend this capability to traditional biomarkers. Presently, conventional practices rely on laboratory-based tests and blood collection in clinical settings, requiring trained personnel and specialized equipment. Real-time, passive/active sensing of physiological and behavioral data, integrated with AI-based models, holds promise for enhancing decision making, diagnosis, and treatment at the point-of-procedure, thus bypassing conventional sampling and in-person tests by scarce expert pathologists in developing nations [18,47]. Digital therapeutics aims to alter patient behavior and address medical conditions using digital technologies. However, its definition often lacks clear criteria that distinguish it from digitized versions of traditional therapeutics. The integration of AI/ML systems to monitor and predict individual patient symptoms employs digital biomarkers in an adaptive clinical feedback loop to offer personalized healthcare. By leveraging AI platforms, tailored therapy regimens can be developed based on diverse personal variables, enhancing treatment efficacy. Furthermore, the combination of digital therapeutics with AI and ML facilitates more efficient clinical observations and management across different health conditions and populations. This unique feature of digital therapeutics enables a personalized approach to healthcare, leading to individual clinical needs, goals, and lifestyles of patients. These attributes are crucial for advancing the field of digital healthcare among patients, physicians, and legislators.

## 3. Monitoring and Detection of Biomarkers with Sensing Platforms

**Figure 3:**
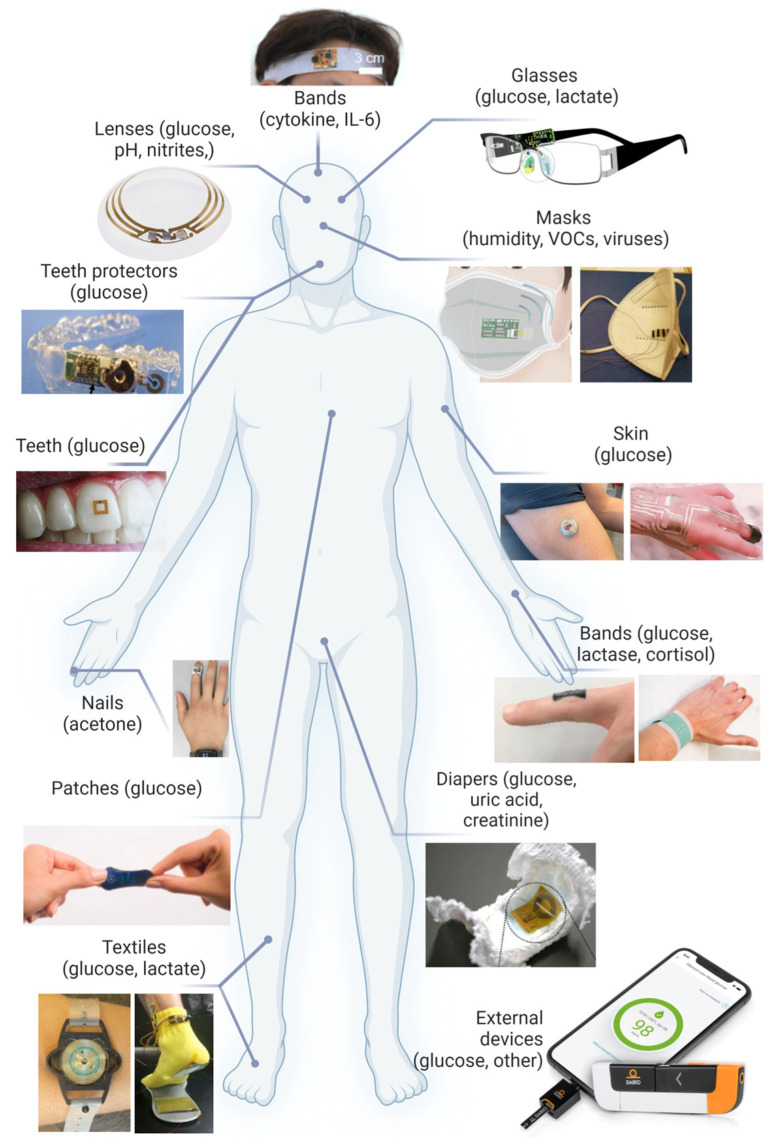
Examples of devices for the detection and/or monitoring of traditional biomarkers.

Blood tests are diagnostic procedures usually applied to confirm the occurrence of particular diseases. They are also helpful in the evaluation of health condition and can support the diagnostic process in patients with cancer [48]. A small blood sample is analyzed for any changes or anomalies in the biochemical status of a given person to indicate a pathological state. Numerous problems, such as infections, autoimmunological diseases, metabolic disorders, cardiological/hepatic disorders, and malignant tumors, can be identified by blood analysis. It can also be employed for monitoring disease progress, the effectiveness of treatment, and identification of potential health problems at their early stage. The blood tests are relatively painless and can reveal important details concerning the health of a particular person. It must be emphasized that the blood tests are not always conclusive and proper diagnosis may require either repetition of the tests or additional investigation procedures. More detailed evaluation of a patient’s health often requires additional tests to be conducted with specialistic equipment and diagnostic methods and techniques, such as medical imaging, gas chromatography–mass spectrometry (GC-MS), MRI, X-ray, and genetic tests. Usually, all these activities call for trained personnel to collect blood samples and carry out the examination using complicated procedures and often expensive tools/tests. Medical imaging is an important diagnostic tool for various disorders, utilizing a range of modalities including X-ray imaging, whole slide imaging, computed tomography (CT), ultrasound, magnetic resonance imaging (MRI), and positron emission tomography (PET). Fortunately, there are numerous publicly available imaging and biological databases offering excellent opportunities for building AI-based systems [49]. Employing AI methods to support pathologists in conventional clinical diagnostics, clinical trials, and translational research enhances diagnostic accuracy, accelerates research insights, and improves patient outcomes through streamlined data analysis and decision support. Moreover, AI-powered applications are designed to enhance care coordination by reducing delays in clinical workflows, using AI to generate and send timely alerts to clinicians, including cancer screening results, diagnostics, prevention, and improved cancer management [50]. On the other hand, the concept of portable or wearable devices, operated by non-experts, enables faster and more frequent testing with reliable and fast results that can provide rapid help in the selection and direction of treatment. This can be beneficial in every configuration, beginning with domestic patients, then emergency patients where doctors must make fast decisions, and finishing with hospitalized patients. The COVID-19 pandemic made everybody aware of the need for fast and continuous health monitoring. Fast access to the test results as soon as the disease symptoms appear calls for a response within a few minutes, which would be an undeniable benefit for public healthcare, for instance, during a pandemic. Hence, during the pandemic, there was a rapid growth in wearable and portable PoC devices for automated, real-time health condition monitoring. The current market of biosensors is worth USD 25.5 billion (2021), and it is predicted that in 2026, it is going to reach ca. USD 40 billion [51]. Smart electronic devices (for instance, smartwatches) can detect the changes in physiological signals, such as pulse rate. Hence, creating digital biomarkers and biophysical stress parameters has gained high acceptance among consumers. Nevertheless, they have no capability of identifying individual biochemical markers (for example, traditional ones), associated with particular diseases, and they cannot be regarded as prognosis tests based exclusively on digital biomarker data. Efforts have been made to develop detection platforms that could minimally or non-invasively detect suitable biomarkers in easily accessible biological fluids, such as saliva, interstitial fluid, sweat, or urine, and simultaneously collect digital signatures [52,53]. Representative examples of the devices employed for the detection of traditional biomarkers are presented in Figure 3.

### 3.1. Eyes, Contact Lenses

Current clinical methods of detection of some vision defects, including cataracts, glaucoma, and red eye syndrome, require the application of such techniques as gonioscopy, pachymetry, perimetry, ophthalmoscopy, and tonometry, which must be supervised by trained ophthalmologists. These techniques are used to detect the actual state of vision defect or require a complex dilated eye examination to diagnose the defect at medium or late stage [54]. So they cannot be employed for diagnostics at early disease stages. This triggered the progress in non-invasive measurements of glaucoma biomarkers, allowing diagnosis at the early stage [55]. Similarly, cataracts can be detected early using smartphone-assisted and ML-supported methods [56]. Moreover, tears can be used for monitoring the physiological state as well as the prognostic factors in serious diseases, such as cancer, Alzheimer’s, and Parkinson’s diseases. Until recently, studies on tear sensors focused on glucose monitoring, but currently, a significant emphasis has been placed on non-invasive analysis of other important biomarkers. The biosensors based on contact lenses constitute an excellent alternative to continuous monitoring of tear components. Contact lenses can be worn without causing eye irritation since they are in direct contact with tears [57]. There are contact lenses integrated with paper microflow sensors for tear analysis, which are capable of detection of hydrogen and nitrite ions, glucose, and L-ascorbic acid using a smartphone [57]. Chromogenic signals from the detection region were scanned by the smartphone and the data were sent to the servers in a cloud using wireless transmission.

### 3.2. Teeth and Mouthguard

Recently, saliva has been extensively utilized in diagnostic tests as an alternative to blood tests. Although progress in electronics and micro-/nanotechnology contributed to the production of miniaturized transducers for fast and sensitive diagnostics, design of a multi-analyte detection platform with high specificity and sensitivity still remains a challenge. Improvements in the field of easily manufactured, multi-analyte, and multiplex biosensors suggest that the development of in vivo sensors and intelligent platforms integrated with AI enables designing and/or mastering such devices in the future. Current clinical standards for the detection of saliva metabolites, such as glucose, are similar to systems for monitoring the blood components. However, glucose content in saliva may not reflect the actual level of glucose in blood, which requires establishing a correlation between the level of glucose in these two fluids. Moreover, some biosensors can detect specific cancer biomarkers present in saliva [58]. After combination with AI, it is possible to obtain clinically relevant information in a simplified way as compared to the standard screening techniques, for instance, colonoscopy or mammography. With contagious diseases, biosensors applied on teeth can be used for their early diagnostics, including COVID-19, HIV, and tuberculosis, via identification of contagious disease factors, such as viruses and bacteria in saliva, commonly carried out by microbiological methods or PCR tests [59,60].

### 3.3. Diapers

Urine is another easily available body fluid that is widely used for the detection of biomarkers, such as glucose, uric acid, volatile organic compounds, red and white blood cells, bacteria, etc. Urine tests are a routine clinical examination, but they require specialists and complicated equipment. Sometimes, urine collection procedures are also troublesome. Routine analysis of carbohydrate content in urine commonly involves strip tests for identification of glucose and ketone levels in patients suffering from diabetes. However, as compared to the analysis of metabolites, fast identification of urinary tract infections remains a big problem, as the classic techniques require several days to identify the bacteria and the level of their sensitivity to antibiotics. Despite intense investigations, the main challenge in the development of biosensors for urinary tract infections is the presence of numerous uropathogens and the increasing antibiotic resistance of bacteria. Recently, biosensor systems were elaborated which couple a diaper with a smartphone application and identify in situ the amount of produced urine to remind the user about changing the diaper and measure in real time the level of biomarkers, including glucose and uric acid [61]. In addition, with the aid of AI and ML algorithms, such biosensors can exhibit superior detection effectiveness and data processing [62]. Clinically relevant data can be extracted and analyzed with two algorithms, random forest (RF) and neural network (NN). A highly sensitive system combined with ML showed a high precision in prostate cancer screening using only one drop of urine.

### 3.4. Wristbands, Headbands, Directly on Skin, Clothes

Wearable sensors are mainly designed to be applied directly on skin [63,64,65,66]. However, there are several examples of clothes-based wearables [67,68]. Also, it is popular to use wristbands and headbands [69]. Such sensing platforms can provide detection of digital and traditional biomarkers. Electrodes monitoring physiological activity, EEG, EKG, EMG, etc., are utilized in clinical practice. Significant importance can be attributed to biosensors applied on the skin, especially for the detection of metabolites present in biological fluids, for instance, in sweat. The problems due to their application result from insufficient adhesion to the skin, skin irritation, lack of comfort, problems with calibration, differences in skin thickness, skin humidity, and others. It is possible to place the sensors directly on the skin using printed temporary tattoos or e-skin or indirectly via plasters/bands, or alternatively via deposition on fabrics with enhanced mechanical resistance to motion [70]. Some applications call for continuous monitoring of sweat’s components, where the amount of sweat produced is insufficient. Furthermore, the result can be affected by analyte dilution upon sweating. That is why a deeper understanding of sweat chemistry and transport as well as intelligent monitoring and prediction are necessary to enhance diagnostic possibilities with this fluid. The solution can be micropump technologies integrated with receptors, which can control the flow of sweat. The newest approach is AI/ML integration aimed at improving the efficiency of the biosensors applied to skin [71].

### 3.5. Face Masks

Analysis of volatile organic compound (VOC) content in breath aimed at diagnosis of various diseases and health conditions originating from specific cells, tissue metabolism, and local microbiome is a field which has dynamically gained popularity. Development works on this subject (generally termed breathomics) have accelerated substantially in recent years. A combination of gas chromatography with mass spectrometry is still a gold standard in the field of analytical approach to the detection of volatile biomarkers. Due to some inconveniences of the classic techniques of volatile biomarker analysis, which involve training of the personnel, cost of sample preparation, analysis, and equipment, a current trend in biosensor development is focused on non-invasive and fast diagnostic tools. In recent years, disease diagnostics has been oriented towards fast, simple, non-invasive methods based on, among other things, the detection of VOCs and their characteristic profiles as diagnostic markers, which are produced by pathological processes changing natural physiological and metabolic routes. There is an increasing interest in the methods for the analysis of exhaled air, which is a multi-component mixture containing numerous volatile substances, for example, aldehydes, ketones, nitrogen oxides, sulfur oxides, and others. Effective identification of the pathological markers at an early stage can provide a diagnosis at the initial phase of a disease, so the patient can be directed to further tests to confirm or rule out the disease. Moreover, breath analysis can also be useful in monitoring respiratory tract inflammation and selecting suitable pharmacological treatments. The low availability of non-invasive identification and monitoring methods, for instance, concerning respiratory tract inflammation, encourages the development of techniques of breath analysis allowing the determination of odor profiles, so-called fingerprints, using electronic and bioelectronic noses (ENs, B-ENs) [37]. It is a dynamically developing field which has clinical potential and can be the source of early detection and evaluation of such diseases as asthma, obstructive pulmonary disease, lung cancer, interstitial pulmonary disease, viral and systemic diseases, etc. [72,73]. Faster diagnosis, for instance, of lung cancer, is highly demanded because the conventional methods—chest X-ray, sputum cytology, biopsy, or computer tomography—fail to provide fast screening tests for large populations. Usually, diagnosis is made at the late disease stage when treatment is already difficult and ineffective. Given the urgency, there is a critical need for effective and fast tools to enable early disease identification through non-invasive breath analysis. Successful implementation depends on progress in biotechnology, micro-/nanotechnology, electronics, supramolecular chemistry, and computational techniques, which improve the metrological parameters of biosensors, so they can be more useful in disease diagnostics. A correlation between variations in breath components and health condition can be monitored in a continuous way, which would have been particularly useful during the pandemic when breath spread the infection. Facemasks with biosensors with porphyrins as the receptor elements were proposed by Di Natale and coworkers. They were able to detect some VOCs absent in normal, healthy breath [74]. A parallel branch of research in the field of biosensors is the elaboration of molecular modelling methods to build a library of receptor elements capable of the detection of selected ligands, which can be classified as volatile biomarkers [37] and be used for PoC test construction. The main advantage of such biosensors is non-invasive sampling and real-time monitoring, which makes them ideal tools from the clinical standpoint. Selected groups of spin-off industrial university companies, including Rapid Biosensor Systems Ltd., Cambridge, UK (rapidbiosensor.com) and Owlstone, Cambridge, UK (owlstonemedical.com), create the breathalyzers, resembling drunkometers, which allow preliminary diagnosis of pneumonia and other numerous pulmonary diseases.

### 3.6. Smartphones

The increasing popularity of health applications for smartphones and peripheral devices such as smartwatches or smart rings offers innovative ways of acquiring and managing data from digital biomarkers [61,75,76]. However, insufficient clarity of their classification, diversity of social groups, and interaction with environmental factors and traditional biomarkers significantly affect clinical translation of those biomedical devices. In a progressively shaping field of acquisition, analysis, and storage of digital biomarkers using smartphones, the key element is understanding its character and capabilities. For example, optoelectronic properties of nanomaterials and nanocomposites can be utilized for smartphone-assisted colorimetric detection of biomarkers [77], a novel procedure involving a non-enzymatic strategy of glucose detection using gold nanoparticles and sensor based on surface plasmon resonance (SPR). This technique was successfully employed for quantitative measurement of glucose in urine. Similar solutions with nanoparticles and smartphone data processing are used for saliva analysis [78]. In addition, the sensor met the PoC test requirements, which makes it a promising platform for biomedical applications in clinical practice. Micro- and nanocomposites with enzymatic properties, due to their high binding specificity and selectivity, exhibit a high PoC test potential, also in combination with wearables. When coupled with suitable AL/ML tools, they can be successfully utilized in the PoC tests, as reported in detail in a review by Jeon et al. [79].

## 4. Cancer Biomarker Detection with Biosensors

The turning points in cancer detection are closely interrelated with the rapid advancements in the field of sensors and biosensors. For example, utilization of microfluidic technology facilitating a precise manipulation of fluids at a micro scale greatly enhances possibilities for reliable cancer diagnosis [80,81]. Furthermore, integration of AI and ML algorithms into medical data analysis has revolutionized the identification of cancer risk factors and the early detection of cancer, offering unprecedented speed and accuracy [82]. In certain biosensors, a substantial volume of data is rapidly generated at the output, requiring additional processing by skilled personnel, which can potentially bring errors. Human-based processing of these data can be time-consuming and significantly hampering the efficiency of the biosensor. Conversely, ML can discern features and trends and offer comprehensible outputs. A cursory web search reveals a remarkable surge in utilization of ML in biosensors over the past decade [83]. Also, AI algorithms have been applied on microfluidics and image cytometry [84,85]. ML algorithms are also employed in analyzing the data obtained by microscopic image cytometry [86]. In the field of cancer detection, electrochemical biosensors are predominant. They encompass various types of biorecognition elements, such as immunosensors, aptamers, enzymes, nucleic acids, etc. [87,88]. These biosensors are favored for their remarkable sensitivity, specificity, cost-effectiveness, and potential for miniaturization. The advancement of various two-dimensional materials, including better synthesis protocols, increased biocompatibility, field enhancement, and an increased surface-to-volume ratio, has accelerated the development of miniaturized sensors aimed at early detection of cancer and other diseases. Moreover, biosensors integrated with nanoparticles offer multiplexing and amplification capabilities [28,89]. Furthermore, wearable electronics [90,91], like electronic tattoos, epidermal electronics systems (EES), and flexible electrochemical bioelectronics, when combined with ML algorithms, offer the capability to monitor various biomarkers in real time, as has been stated. However, most wearable biosensors can detect a small number of biomarkers only. It is needed to develop novel biosensors to detect and monitor a larger range of biomarkers. Understanding the composition of body fluids, as well as their relationship to specific medical diseases, is crucial to gaining general clinical adoption of wearable technology in healthcare [92,93,94]. In the actual world, rigorous and repeatable interpretation of biosensor results is also a goal, especially in applications that may require a clinical or operational reaction. The advancement of diagnostic devices invariably requires assay developer researchers to serve as intermediaries, connecting both ends. Their role involves identifying detection strategies aligned with clinical requirements by comprehensively understanding (1) the intended application of the technology and its fundamental principle, and (2) the preferred test type, be it qualitative or quantitative, addressing challenges related to sample matrices, defining biomarker threshold (cutoff value), and determining whether the system requires a mono- or multiplex assay format [32]. Noninvasive testing with wearable devices is currently limited to a number of metabolites and electrolytes and should be extended by employing more multiplex assays based on biosensors. Various ranges of disease biomarkers, hormones, and stress markers should be employed to the scope of detection. Moreover, apart from conventional fluid types, the potential of novel fluid types such as urine, breath, mucus, and semen should be investigated. Furthermore, various fields of biomedicine, including the clinical development of new experimental medicines guided by biomarkers, will benefit from this real-time examination of a broader spectrum of biomarkers.

The preprocessing of signals generated by sensors and biosensors involves several crucial steps. Initially, data cleaning is conducted to address missing data, outliers, and noise present in the sensor readings. For example, a significant noise interference reduction and enhanced accuracy when measuring glucose levels has recently been reported by Yang et al. [95]. AI-based algorithms, such as those used in glucose prediction and calibration, hold significant potential for advancing continuous glucose monitoring sensors and can be adopted to different biomarkers [96]. Techniques such as mean imputation, interpolation, or specialized ML algorithms designed for handling missing data can be employed to address missing data [97]. Subsequently, data normalization or scaling methods, such as min-max scaling or z-score normalization, are applied to handle variations in scales and ranges observed in sensor data, which can impact the performance of machine learning models. These techniques ensure that no single feature dominates the learning process by bringing all features to a similar scale. Moreover, feature selection or extraction methods are utilized to identify the most relevant features from a potentially large number of features present in sensor data. Commonly used techniques for this purpose include correlation analysis, mutual information, or feature importance measures. Finally, the preprocessed sensor data are partitioned into training, validation, and testing sets. The training set is used to train the machine learning model, the validation set aids in tuning hyperparameters, and the testing set is employed to evaluate the final performance of the trained model. Advancements in AI-based algorithms offer promising potential for enhancing sensor performance by facilitating rapid sensor design and automated data processing, thereby enabling them to effectively address future challenges [98]. Additionally, integrating physical knowledge can enhance algorithm performance and alleviate optimization difficulties. For instance, Khatib et al. [99] found that incorporating knowledge of the underlying physics as input and pre-training these quantities during the training process holds promise for improving network performance and mitigating challenges associated with sensor optimization. Moreover, elaborated Mean Squared Error calculated for the cross-validation set after training serves as a valuable measure for evaluating the model’s prediction accuracy. Hollon et al. developed an optical imaging technique employing the CNN algorithm to predict diagnostic test results [100]. Lussier et al. established a 1D CNN model for assessing chemical spectra in multiplexing SERS sensing [101]. Moreover, the ML algorithms can facilitate prediction or decision making from diverse digital data sources [102]. The concept of the digital twin originated in the healthcare industry for product or equipment prognostics [103]. Angulo et al. proposed a versatile framework for developing digital twins applicable to healthcare, particularly for lung cancer patients [104]. Digital twins represent a cutting-edge approach in digitalization across industries, attracting interest from two key groups: (i) data analysts, tasked with developing expert recommender systems and extracting knowledge through explainable AI, and (ii) medical professionals, who leverage this knowledge to enhance diagnostic capabilities. The resulting software platform holds potential as a versatile service tool applicable across various fields of expertise, with particular relevance in healthcare and industry sectors. Laubenbacher et al. explored the use of medical digital twins to combat COVID-19 infections and future pandemics, emphasizing their potential to optimize treatment through a combination of mechanistic understanding and AI techniques [105].

Recent research indicates that electrochemical sensors and biosensors have emerged as powerful instruments to gain comprehensive understanding and detection of disease-associated biomarkers. Substantial progress in nanomaterial and biomolecule methodologies, aimed at enhancing sensitivity, has led to the creation of electrochemical biosensors capable of real-time detection of single and multiple biomarkers in clinically relevant samples, which was comprehensively presented by Kim et al. [106] and more recently by Jarahi Khameneh et al. [107]. The review by Sinha et al. [108] was focused on chronic disease biomarkers detected through electrochemical sensors and explores the potential of artificial neural networks (ANNs) for disease monitoring. Additionally, it discusses risk factors, causes, and severity of chronic diseases, and how ML algorithms can utilize biomarkers and clinical symptoms for analysis. Finally, it highlights the use of ANN to predict and diagnose chronic diseases, offering insights for the future development of innovative analytical tools in healthcare. Some of the most recent concepts are presented below. The biomarkers produced during metabolic processes are becoming increasingly important for the early detection of diseases. However, detecting only one analyte has its limitations, as it may be associated with various conditions. Therefore, in the case of disease monitoring, which typically arises from the presence of multiple complications, multi-analyte sensing platforms are essential for accurate diagnosis [109,110].

Electrochemical aptasensors are frequently employed in the detection of cancer biomarkers. These sensors can be categorized into three groups for detecting cancer biomarkers: those designed to identify exosomes, circulating tumor cells, and protein tumor biomarkers. Addressing the need for a sensitive and convenient diagnostic tool, Hou et al. [111] developed a platform that can directly and accurately detect target miRNA-21, a breast cancer biomarker, in serum without sample preparation and purification. This aptasensor comprises gold nanoparticle-coated microgel particles with a porous network structure, mimicking the biocompatible microenvironment of biological tissue for enhanced RNA detection. Covalently immobilized amino-modified oligonucleotide chains serve as capture probes complementary to miRNA-21, enabling monitoring of miRNA-21 hybridization via differential pulse voltammetry (DPV). The aptasensor exhibits a linear detection range from 10 aM to 1 pM, with a detection limit of 1.35 aM. This approach has promising potential for early diagnosis and treatment monitoring in breast cancer patients. A novel one-step multiplex analysis of breast cancer exosomes using an electrochemical strategy assisted by gold nanoparticles was presented by Zhang et al. [112]. The aptasensor utilizes a multi-probe recognition strategy, incorporating CD63, HER2, and EpCAM aptamers as capture units, along with methylene blue (MB) and ferrocene (Fc) functionalized aptamers as signal units. Importantly, the method demonstrates the ability to distinguish between different types of breast cancer exosomes, including HER2-positive and HER2-negative subtypes, and exhibits compatibility with complex sample matrices, offering promising prospects for the screening and prognosis of breast cancer by exosome analysis.

**Figure 4:**
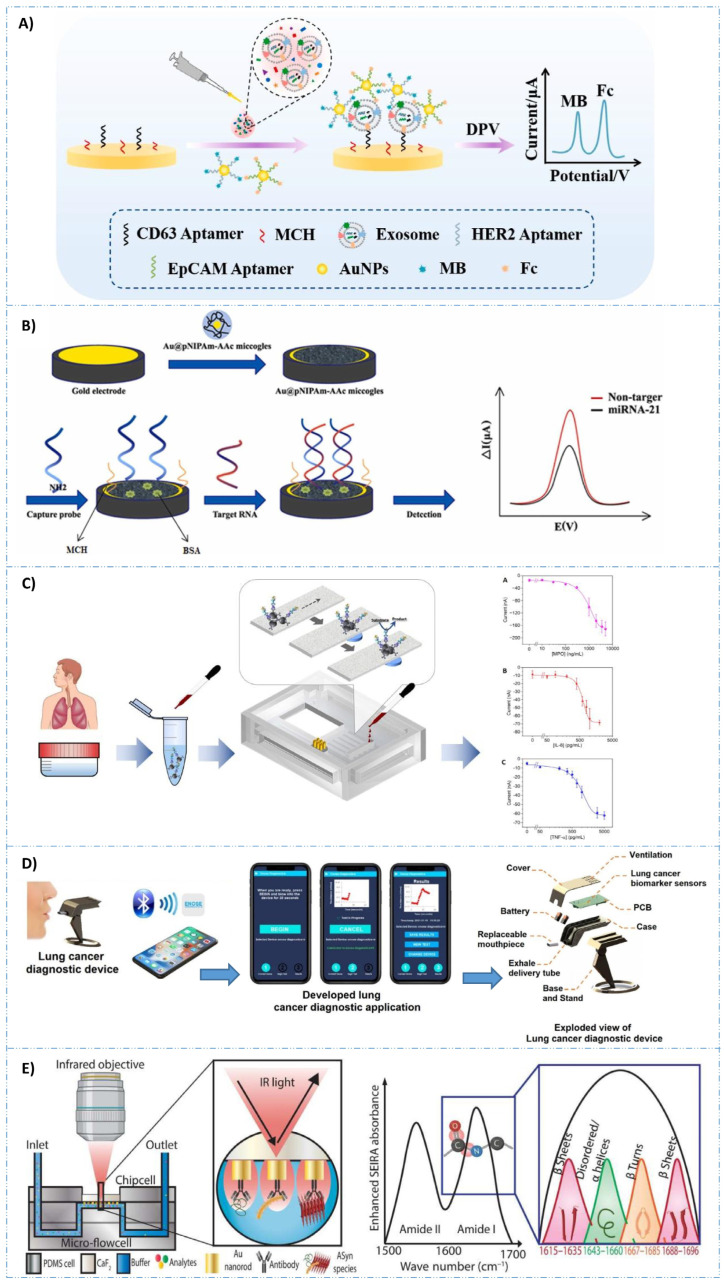
(**A**) One-step multiplex analysis of breast cancer exosomes based on an electrochemical strategy assisted by AuNPs. Reproduced with permission from [112]. (**B**) Setup of the AI–coupled plasmonic infrared sensor for the detection of structural protein biomarkers in neurodegenerative diseases. Reproduced with permission from [130]. (**C**) Scheme of the multiplexed quantitative detection of biomarkers in sputum by a PoC paper-microfluidic electrochemical device [113]. (**D**) Example of a handheld LC diagnosis device based on MIP sensor. A patient blows into the replaceable mouthpiece and the results will be shown on his/her smartphone instantly. The mobile application that graphs the data during the test, and the exploded view of the proposed lung cancer diagnosis handheld device. Reproduced with permission from [114]. (**E**) The construction and working process of the AuNPs@NIPAm-co-AAc microgel electrodes and detection process of miRNA-21. Reproduced with permission from [111].

A novel multiplex device integrating paper-microfluidic technology, electrochemical transduction, and magnetic nanoparticle-based immunoassay was developed by Gutiérrez-Capitán et al. [113] for simultaneous detection of interleukin-8 (IL-8), tumor necrosis factor-α (TNF-α), and myeloperoxidase (MPO) biomarkers in sputum. The device features an on-chip electrochemical cell array and a multichannel paper component. With its potential for low-cost mass production, this device offers promise as a PoC solution for improving diagnostics and advancing personalized medicine. There is a high demand for devices that enable early diagnosis, minimum costs, and time of assessment. Recently, a novel handheld electronic device for early LC detection by analyzing exhaled breath was presented by Emam et al. [114]. Utilizing an electrochemical gas sensor with a graphene and Prussian blue layer on a chromium-modified silicon substrate, the device employs MIPs for selective biomarker binding. The device’s efficacy is demonstrated through its ability to detect biomarker concentrations at the 1–20 ppt level. Equipped with a printed circuit board for resistance measurement and Bluetooth connectivity for data transmission to a smartphone app, this device offers promising potential for non-invasive LC diagnostics (Figure 4). Advancements in deep learning (DL) and AI have facilitated the classification of pattern data from larger sensor arrays. Furthermore, the development of artificial olfactory sensor technology has seen notable progress with the integration of ANNs [115]. The artificial olfactory sensor system enables the analysis of chemical composition and the quantitative and qualitative levels of trace volatile organic compounds, required particularly in biomarker detection. By integrating IoTs with volatile biomarker detection, it is envisioned that these sensors will become commonplace in mobile wearable devices, seamlessly integrating into our everyday routines. In the near future, monitoring VOCs may become a substitute to conventional medical diagnostics.

Cancer biomarkers can be identified through biorecognition elements such as peptides, proteins, DNA, enzymes, and aptamers [116,117]. Electrochemical biosensors (EBs) can be categorized into immunosensors, aptasensors, enzymatic biosensors, and nucleic acid biosensors, depending on the specific biorecognition element employed [42]. There is also an interesting trend of combining AI and ML with standard techniques, like NMR, MS, and IR spectroscopy [118]. Such integrations have demonstrated potential in various fields including environmental monitoring [119,120,121], food chemistry [122], chemical sensing [123,124], biosensing [125], diagnostics [126,127], etc. [128]. Moreover, AI has been integrated with surface-enhanced IR absorption (SEIRA) spectroscopy, allowing for dynamic monitoring of protein interactions with other biomolecules such as lipids, nucleic acids, or carbohydrates, especially when they are present simultaneously [129]. Recently, Kavungal et al. integrated AI with SEIRA to solve a more intricate challenge, distinguishing between various aggregation states of a particular protein within a mixture [130]. In conclusion, they demonstrated a structural biosensor capable of extracting the distinctive absorption signature of pathological protein biomarkers from the intricate biomatrix of human cerebrospinal fluid (CSF). This advancement brings us closer to broadening the application of sensors for diagnostic purposes in neurodegenerative diseases (NDDs) within clinical settings.

Over the past few decades, achieving early and precise cancer detection has emerged as a critical goal to enhance survival rates and patients’ quality of life. The integration of state-of-the-art technology, AI, and data analysis has expanded the field of oncology, opening new pathways for early detection and characterization of diverse cancer types. Talens et al. [131] aimed to create a groundbreaking diagnostic tool utilizing EN technology and AI for non-invasive prostate cancer detection. The authors have successfully engineered a robust neural network tailored for prostate cancer detection, using MOOSY-32 EN technology and advanced AI methodologies. This innovation has the potential to boost advancements in early disease detection, facilitating more accessible and efficient diagnostic tools across various clinical and medical applications. The ways in which data processing and ML techniques can be used to facilitate the use of electronic olfaction and gustation in the detection of disease have recently been provided by reviews [132,133,134].

An increase in the popularity of DL and ANNs can be seen, with a majority of recent publications favoring these methods. This is due to the fact that those are the most widely known techniques for researchers working in fields related to biosensors. An intriguing finding is that biosensors employing electrical detection techniques seldom utilize DL as the analytical tool for classification. This could be attributed to the data-intensive nature of DL, coupled with the deficiency of established databases for biosensing data. Conversely, there exists an abundance of readily available datasets suitable for training samples in image and optical detection of various biomarkers. In summary, the integration of ML algorithms into biosensors offers significant advantages by automating the tasks of data extraction, processing, and analysis. Such automation obviates the need for an experienced professional to interpret the data, bringing us closer to delivering PoC healthcare solutions in resource-constrained environments.

## 5. AI-Assisted Diagnosis

Multiomic investigations, owing to precise and accurate results for a particular person or a larger group of people, are gaining increasing attention among scientists and clinicians. An advantage of multiomic technologies in genomics, transcriptomics, epigenomics, proteomics, metabolomics, and other fields is believed to be the key to the development of personalized medicines tailored to a particular patient [135]. For instance, there are attempts to generate complex multiomic profiles for 20,000 entities and additional data for different cancer types in the coming years (The Cancer Genome Atlas—TCGA. However, as compared to the conventional multiomic analysis producing a large volume of data in a single experiment that is not conducted in real time, multiomic approach-based biosensors can become promising, cost-effective platforms for real-time monitoring. Simultaneous monitoring of many biomarkers via multiplexing of the biosensors can lead to early identification, prevention, and treatment of diseases in real time [136]. In this regard, the studies on versatile bioelectronic/electrochemical sensors for simultaneous detection and continuous monitoring of different biomarkers using simple, inexpensive, and fast-responding elements can provide personalized diagnosis and fluent clinical transition. Nevertheless, analysis of the data generated by many systems calls for an advanced computational approach, beginning with data integration, statistics, and AI/ML systems, to become useful for patients and doctors. Scientists place the spotlight on multiplexing of electrochemical biosensors, whose effectiveness can be substantially increased after integration with well-trained AI and/or ML to arrive at clinically significant results. AI/ML can be used to sort and predict the results from biosensors as well as to increase their sensitivity. Hence, it is important to understand and develop AI/ML to take full advantage of the biosensors’ potential in the clinical field. Although the integration of AI with biosensors still needs a lot of effort and improvement, intelligent data processing can revolutionize the field of biosensors. In recent years, wearables supported by AI appeared as an inexpensive solution for monitoring health condition. AI explores available data to search for digital anomalies, sort data, reduce noise, integrate bio-digital markets, and draw clinically relevant conclusions. To achieve the desired precision, AI plays two important roles. The first focuses on reducing undesired information before data transmission, which at the same time reduces the consumption of energy necessary for wireless transmission. The other role is to emphasize data quality parameters, such as accuracy, repeatability, and stability. These aspects are important for investigation of the interrelation between the biomarkers in complex biological samples as well as in the case of traditional and digital markers, offering a perspective for the solution of urgent challenges in the field of biosensors, such as accuracy, reliability, response time, and lifetime [137]. For example, Raman optical imaging combined with deep convolutional neural networks (CNN) was elaborated for the automated prediction of a brain tumor in real time [100]. The prediction based on CNN revealed a 94.6% accuracy, which was higher than the one from the interpretation of conventional histologic images by the pathologists (93.9%). Similarly to CNN, ANN, recurrent neural network (RNN), support vector machine (SVM), principal component analysis (PCA), hierarchical clustering analysis (HCA), decision tree (DT), partial least squares discriminant analysis (PLSDA), or partial least squares regression (PLSR) are available and accepted for biosensor applications.

In the field of health monitoring and disease diagnosis, the emergence of AI offers robust tools and algorithms for data processing and analysis, addressing the developmental constraints encountered by health monitoring sensors [138,139,140]. Through AI algorithms, signals and data regenerated by sensors can undergo sophisticated processing and analysis, thus facilitating intelligent health monitoring. In particular, the wearable intelligent systems featuring tailored structures and compositions as well as enriched functions enable human beings to access a next-generation closed-loop platform for early disease prevention and diagnosis [141]. Such systems, combined with ML algorithms, can sift through vast datasets to unearth potential health issues, furnishing doctors and patients with more precise diagnoses and treatment strategies. According to Göndöcs et al. [142], AI algorithms can be useful to support decision-makers, should not replace decision-makers. Decision making uses algorithmic analysis, but it is not solely algorithmic analysis; it also involves other factors, many of which are very human, such as creativity, intuition, emotions, feelings, and value judgments. AI algorithms can also be used in inverse design, leading to a reduction in the volume of training data by utilizing both labeled and unlabeled data [143].

There are common challenges encountered in any ML application regarding both the data utilized and the model itself [144,145]. Developers of ML applications for sensing devices must address challenges regarding:

- data availability;
- model selection;
- reliability;
- deployment alternatives;
- security and privacy;
- utility and user acceptance;
- communication;
- power consumption limitations;
- storage limitations.

Certain matters can be addressed through clinical and preclinical investigations to ensure an appropriate user interface and documentation regarding the confidence or dependence on the results, as required by regulations. The ML model must be deployed and utilized in both retrospective and prospective studies, with the clinical impact assessed [146]. In many healthcare applications, it is advantageous for the ML models to be customized according to signals from individual patients. This approach enables each device to train a personalized model based on the user’s data, leveraging both cloud-based and local data [145]. Efforts to minimize power consumption in data transmission and reception have taken various approaches. These include the development of dedicated embedded hardware to execute ML algorithms, minimizing the volume of data transferred, employing data compression techniques, scheduling data transfer, computational offloading, and the creation of self-powered devices [66,147,148,149,150].

ML can be realized in either a supervised or unsupervised way. The supervised algorithm was utilized, among other purposes, for precise prediction of the glucose level in blood based on analysis of VOCs in exhaled air [151]. Apart from the analysis of glucose in the blood, SVMs are widely used for the diagnosis of cancer and identification of pathogens transmitted in water. SVM for breast cancer increased the accuracy of diagnosis by 33.34% and reduced variance of diagnosis by 97.89% [152]. Unsupervised ML algorithms are employed for the analysis and grouping of unmarked datasets [153]. These algorithms can reveal hidden patterns within data without human intervention. PCA is an example of an unsupervised ML algorithm used for dimensional reduction via substitution of a set of variables with the principal components and it is widely utilized in sensor and biosensor systems [154,155]. There is extensive literature on biosensors coupled with AI/ML [90,140], presenting preliminary investigations waiting for clinical evaluation. For example, much research has been conducted on clinical patient data used for optimization and control of the measurement algorithm for wearable skin biosensors for glucose control. Diagnostic results from the trained algorithm are comparable to the glucose concentration in blood measured using the reference method and simultaneous measurements of heart rate and SpO2. Nevertheless, those algorithms have certain limitations, especially prediction algorithms, which are generally designed to improve the precision of the devices but typically rely on calibration using gold standards. Owing to these technologies exhibiting acceptable levels of accuracy with well-suited algorithms, their application as diagnostic tools will certainly rise in the near future, as shown by intensified research on this subject [96].

**Figure 5:**
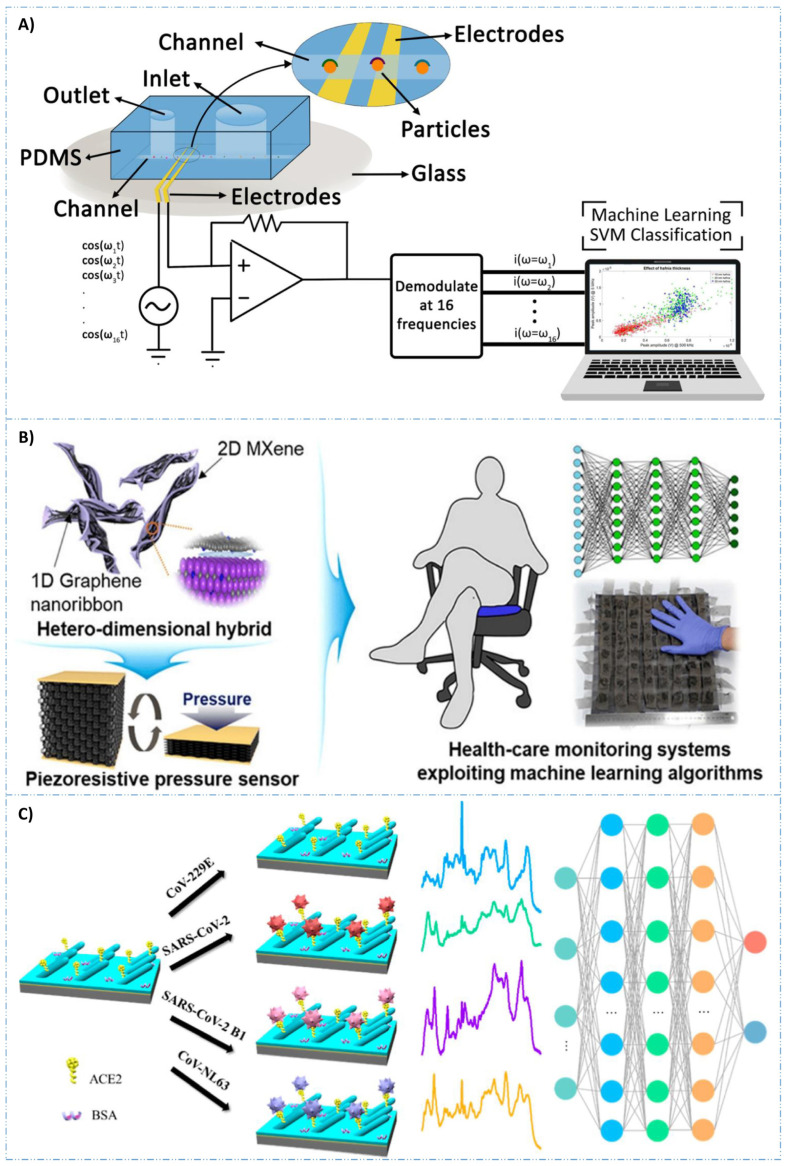
(**A**) Scheme of electrical impedance cytometer. As cells pass from the inlet to the outlet in these biosensors, alterations in impedance are detected by a lock-in amplifier. This amplifier can simultaneously apply signals at various frequencies. Subsequently, the data are recorded and analyzed using SVM. Reproduced with permission from [155]. (**B**) Interfacing 1D graphene nanoribbons with 2D MXene for the development of pressure biosensor, trained using ML algorithm. Reproduced with permission from [157]. (**C**) Schematic illustration of angiotensin converting enzyme 2 (ACE2)-functionalized AgNR@SiO_2_ array for SARS-CoV-2 variant detection. Reproduced with permission from [162].

Despite recent advancements, significant challenges remain in achieving commercial maturity for AI biosensors in IoT applications [16]. Crucial to these applications are flexible bioelectronic materials that seamlessly integrate with the human skin. Present soft wearables mainly capture physiological signals and transmit them to external computing devices. Moreover, the use of multi-analyte detection approaches and biocompatible materials opens new avenues for developing electrochemical sensors designed for wearable detection of diseases [17]. Flexible bioelectronics provide mechanical flexibility akin to human organs like skin and muscles, reducing tissue damage and long-term adverse effects. Electrical detection uses circuits to capture biosignal data as electrical signals. Impedance is typically used to identify and quantify cells, based on changes as the cells pass through microfluidic electrodes. This signal reflects cell properties such as size, conductivity, and permittivity. As compared to traditional optical methods, electrical detection offers advantages such as smaller size and lower cost owing to the absence of bulky optical equipment. Figure 5A illustrates a schematic diagram of an electrical impedance cytometer with SVM for data analysis [156]. Creating hybrid nanocomposite materials by combining 2D nanostructures, e.g., MXenes, borophene, etc., to enhance biosensor parameters has also been attempted [157,158]. The interfacial integration of 2D materials with 1D graphene nanoribbons has been explored to develop a pressure sensor with an improved life cycle. ML approaches were utilized to train the sensors to detect various sitting postures with over 95% accuracy (Figure 5). Carbon-based nanomaterials, including carbon nanotubes and graphene, retain their intrinsic electrical properties and exhibit excellent biocompatibility, making them ideal for bio-signal monitoring [159,160,161]. These materials facilitate integration with skin-compatible devices to develop wearable monitoring systems (Figure 5B) [162]. An integrated approach combining surface-enhanced Raman spectroscopy (SERS) with a specialized DL algorithm, CoVari, was proposed [163]. This systems’ ability to predict both viral species and concentrations simultaneously, together with its potential for PoC diagnostics, underscores its novelty and broad applicability in virus detection (Figure 5C). Moreover, implementing a smart sensor system that relies on large datasets and advanced algorithms is challenging, particularly regarding data processing and storage. Recently, cloud computing has become the preferred method for processing sensor signals due to its powerful computational capabilities and vast storage [164,165]. Integrating cloud technology with biosensors is common in monitoring applications with expanding data volumes. The essence of mobile edge computing lies in transferring some or all computing tasks from the original cloud computing center closer to the data source. This approach holds significant potential for addressing the limitations of sensor–cloud systems. This approach offers benefits such as improved computational efficiency, faster network processing, and cost-effectiveness. Consequently, advanced biosensors are likely to increasingly utilize this technology. A key challenge in deploying AI-powered biosensors is ensuring unbiased outcomes. ML algorithms can exhibit disparities among different population groups, especially those already marginalized [166]. To address this, several strategic measures are needed in the development of ML applications utilizing biosensors. These include incorporating diversity in data collection and establishing robust post-application performance audits to assess the impact on vulnerable communities. Technically, monitoring model performance and logging for performance drift detection are crucial [167]. Implementing these procedures is essential for building confidence among healthcare professionals and patients in the provided services.

A comparison of different developed biosensors with ML analysis for cancer detection was recently presented by Kokabi et al. [137]. Table 1 presents an overview of other examples of applications for sensor devices integrated with ML algorithms.

## 6. AI-Assisted Biomarker Discovery

Recently, advancements in healthcare digitization and personalized treatments have led to groundbreaking developments. Utilization of AI and ML has the potential to enhance comprehension of disease onset and progression, potentially uncovering new disease subtypes [184], unveiling novel drug targets [185], advancing the field of precision medicine [186], propelling efforts towards disease prevention by providing insights into preventive strategies [187], and finally, discovering new biomarkers for the diagnosis of diseases [188].

Various biological samples can be utilized to detect disease indicators, biomarkers. According to the National Institutes of Health (NIH) and the Food and Drug Administration (FDA) [189], the basic definition of a biomarker is ‘A defined characteristic that is measured as an indicator of normal biological processes, pathogenic processes or responses to an exposure or intervention’. As outlined by the US Food and Drug Administration (FDA), biomarkers can be divided into seven distinct categories: susceptibility, prognostic, diagnostic, prediction, monitoring, response, and safety. By grasping these classifications, it becomes possible to adopt standard protocols by supporting a biomarker with its specific function and context, thereby enhancing the efficiency of developing accurate diagnostic and treatment methods. Consequently, this approach aids in the advancement of innovative strategies and instruments for the discovery of new biomarkers [190]. This expansive definition allows for their application in numerous areas such as diagnostic, monitoring, pharmacodynamic/response, predictive, and prognostic biomarkers [191]. While invasive sampling methods often cause discomfort, leading to potential patient aversion, constraints in sample availability and frequency, and higher costs, non-invasive methods alleviate many of these issues. However, the availability of non-invasive samples like urine, feces, and sputum is finite, and their collection may be hindered by the patient’s feelings of embarrassment or discomfort [192,193]. Biomarkers offer a deeper insight into the disease’s progression and treatment outcomes than do conventional health indicators. Artificial intelligence technologies represent advanced approaches that may optimize the potential for discovery of various biomarkers [19,188,194,195,196,197].

Clinical researchers are constantly on the lookout for novel biomarkers and have lately turned their attention towards digital, uncommon markers. These digital biomarkers typically merge biological, neurological, socioeconomic, and environmental data, forming an intermediate biomarker [198]. Recently, the use of ML and DL methods has been increasingly popular for biomarker discovery. Both supervised and unsupervised learning approaches are employed to unearth biomarkers from a variety of biological data. Supervised learning utilizes methods such as RF, SVM, Logistic Regression, along with DL techniques like CNNs and RNNs. On the other hand, unsupervised learning employs PCA, ICA, and clustering algorithms to uncover novel biomarkers [199]. The integrative digital biomarker will prove more beneficial for researching diseases that necessitate the combined analysis of data from various sources. By ensuring no subtle patient signals are overlooked and taking into account the interplay between different signals, this approach will aid in prompt detection and more precise forecasting of symptoms [200]. Progress in Next-Generation Sequencing [201] and our comprehension of the human genome have transformed biomarker discovery, especially in oncology [202]. Conventional biomarkers typically relied on circulating markers in blood, plasma, and serum, or those detectable through imaging methods. However, the emergence of genomics has allowed for deeper exploration, identifying individual genetic variations that significantly influence disease pathology, particularly in cancers where genetic mutations frequently dominate [203]. AI-based tools demonstrate significant improvements in oncologic clinical trials, with a 50% increase in identifying potentially eligible patients and a 25% reduction in time for patient screening [204]. A diverse variety of molecular, histologic, radiographic, or physiological entities or features are common types of cancer biomarkers. Recent substantial advancements in methodology and insights have propelled significant progress in the field of discovery of biomarkers. The AI/ML-based tool Excelra is dedicated to personalized medicine and biomarker identification [205]. Within the oncology domain, an internal ML model harnesses expression data from CCLE, COSMIC, and ArrayExpress, employing RF regression and recursive feature elimination (RFE) with ridge regression for feature selection. Trained with SVM and RF, the model accurately predicted drug response for nine out of ten patients, achieving an 82% accuracy in identifying drug response biomarkers.

While AI-powered applications hold considerable promise, further research is essential to validate these tools and streamline their adoption. The importance of biomarkers in clinical evaluations has been well recognized, yet identifying novel, specific, and single-molecule biomarkers remains a challenging task. This attempt necessitates a deep understanding of a disease’s biological mechanisms and the effects of new drugs. The complexity of most diseases, which can vary widely based on an individual’s health conditions, lifestyle, and diet, adds to this challenge. For instance, glucose serves as both a diagnostic and monitoring biomarker for diabetes but may also indicate stress or other health issues. Similarly, electrolyte imbalances could point to dehydration, hyperkalemia, kidney diseases, and various other conditions. The multitude of metabolites and small molecules associated with different health conditions emphasizes the critical role these substances play in bodily functions. Consequently, pinpointing precise biomarkers is difficult, as any newly discovered compound could be linked to multiple known or unknown conditions. However, the process of systematically testing each biomarker’s concentration for every disease proves to be both costly and time-intensive. Chemical sensors and biosensors present a promising solution to the challenges of biomarker discovery by enabling the simultaneous tracking of a wide array of molecular signatures for comprehensive multiomics analysis [206]. Their capacity for continuously monitoring and detecting time-sensitive patterns offers significant advantages in identifying biomarkers that exhibit rapid fluctuations over short durations. Potential applications include wearable biosensors for tracking cardiac health, epilepsy, or other sudden medical conditions, providing insights into the body’s physiological changes preceding such events. Currently, no devices or tests can foresee critical incidents like heart attacks, epilepsy seizures, or heatstrokes. Furthermore, many health issues, such as Alzheimer’s disease or long-term effects of COVID-19, lack established early warning biomarkers [207]. The continuous, real-time tracking of various biomolecules during everyday activities could yield critical insights into the biochemical markers of these abnormal conditions. This capability enables swift analysis of molecular signatures, and when combined with data analytics, it enhances the biomarker discovery process in ways that other analytical methods cannot match. However, monitoring of a single biomarker for disease diagnosis has shown to be inadequate, as it is often influenced by a mix of other chemical and physical markers. While various models have been suggested for conducting multiomics analysis [208], they typically depend on molecular data gathered through diverse techniques at separate times, leading to increased inaccuracies. Recent advancements in miniaturization of biosensors have facilitated the inclusion of multiple sensors within a small area. This progress has led to the creation of sensor arrays capable of analyzing various analytes from a single biofluid sample, thereby opening new possibilities in chemical biomarker discovery through multiplexed sensors that can track numerous analytes at once, in real time [209]. Continuous monitoring of heart and respiratory signals, such as cough frequency, body temperature, and movement patterns, has proven effective in identifying early signs and progression of COVID-19 [210]. Sensors that concurrently assess both chemical and physical health indicators are poised to provide a more detailed and holistic view of an individual’s health status, enhancing the precision of diagnoses. These integrated devices, capable of simultaneous monitoring of chemical analytes and physical parameters, represent a significant leap forward in the field of health monitoring and diagnostics. Recently elaborated databases identifying VOC signatures from specific bacterial species, microbiomes, and exhaled breath from patients with diseases, such as respiratory tract infections, gastrointestinal conditions, and inflammatory syndromes (e.g., acute respiratory distress syndrome and sepsis), offer scientists new opportunities to discover biomarkers for diagnosing and triaging various diseases.

**Figure 6:**
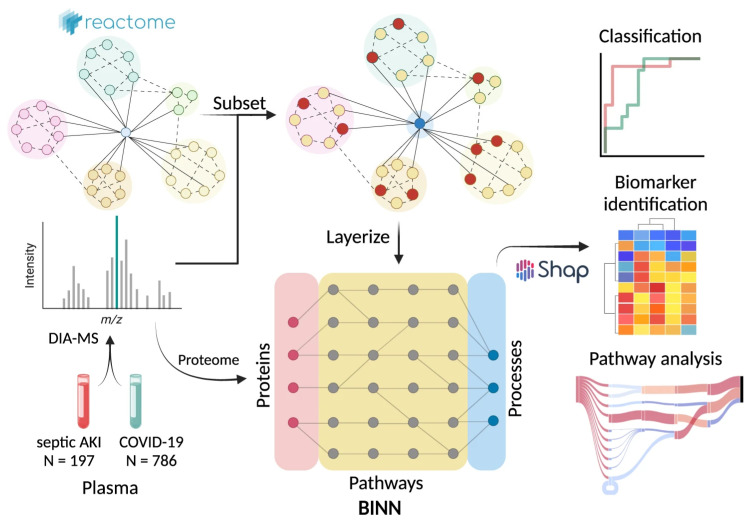
The scheme of analyzing proteomic data using BINNs. First step is the creation of a BINN for each dataset by selecting relevant pathways from a database such as Reactome. BINNs are trained using protein quantities from each sample to distinguish between two subphenotypes. Subsequently, SHAP (feature attribution method) is used to interpret the networks, providing feature importance values for biomarker identification. Reproduced with permission from [213]. Created with BioRender.com.

To understand disease mechanisms and the identification of biomarkers, analyzing the proteins and related biological pathways of a disease is crucial. Integrating ML techniques into proteomics processes enhances the detection of disease-related biomarkers and biological pathways [211,212]. Nonetheless, models like deep neural networks (DNNs) often lack interpretability. Hartman et al. recently proposed a DL strategy based on biologically informed neural networks (BINNs) that merges the analysis of biological pathways with biomarker identification to enhance the interpretability of proteomics experiments [213]. The presented software has shown the capability to explore complex biological systems more thoroughly and enhance the prospects of biomarker discovery in proteomics (Figure 6).

Innovations in (bio)sensor technology, specifically in multimodal and multiplexed sensors, have enabled significant advancements in the continuous collection of detailed multiomics data related to patients’ health from biofluid samples [214]. These datasets consist of complex, multivariable, and non-linear patterns that traditional analysis methods struggle to handle. In healthcare, ML has proven to be a powerful tool for making sense of large and complex datasets. The role of ML algorithms in enhancing the transmission of heart rate data in terms of both accuracy and efficiency, focusing on time series healthcare metrics, has been proven [215]. Advancements in chemical sensing technology have improved the sensitivity and selectivity of detecting targets, leading to new insights into how chemical biomarkers are linked to specific diseases [155,216]. Among the primary types of ML algorithms, supervised learning has been pivotal in identifying relationships among molecular analytes, vital signs, and health outcomes. Specifically, SVM models have been effective in drawing connections between VOCs in breath and blood glucose levels, achieving up to 97.1% accuracy in classifying artificial breath samples against known glucose levels using diabetic breath analysis data [151]. The development of more robust sensors, combined with SVM models, promises the discovery of real-time links between clinically validated biomarkers and the analytes measured in non-invasively collected body fluids. Sensors, powered by ML, hold the potential for ongoing monitoring of a wide array of biomarkers, providing continuous feedback on an individual’s metabolic and immune states through the chemical composition of interstitial fluid, sweat, or saliva. Furthermore, analyzing multiple biomarkers across various body fluids can reveal associations with clinically significant conditions. ML is a versatile tool, enabling not only the prediction of events but also the identification of those already underway. This capability is particularly useful in monitoring epileptic patients, where a combination of sensors measuring galvanic skin response, heart rate, temperature, and movement can pinpoint ongoing seizures and notify caregivers [217]. Beyond the realm of supervised learning for regression and classification, unsupervised ML techniques are valuable for uncovering hidden features in unlabeled molecular sensing data. These techniques facilitate the creation of simplified data representations and spotting of outliers within data clusters, using methods such as k-means clustering. With the thorough analysis of data from biosensors, reinforcement learning algorithms can be incorporated to provide medical guidance and prompt interventions [108,218].

Integrating multiplexed, multimodal, real-time chemical sensors into the big datasets can unveil novel connections between established biomarkers and emerging chemical patterns, enabling ongoing monitoring via readily obtainable body fluids. The fusion of these vast data streams from sensors, which track both chemical and biophysical indicators, with ML methodologies, is expected to significantly advance biomarker identification and, more broadly, transform healthcare practices. Developing a comprehensive platform that consolidates chemical and biophysical data from various sensor technologies is crucial for evaluating and confirming the relevance of newly identified biomarkers to specific health conditions. In essence, the incorporation of fresh chemical insights from alternative bodily fluids, as provided by real-time monitoring sensors, will greatly enhance the diagnosis and prevention strategies for a wide array of medical conditions.

**Figure 7:**
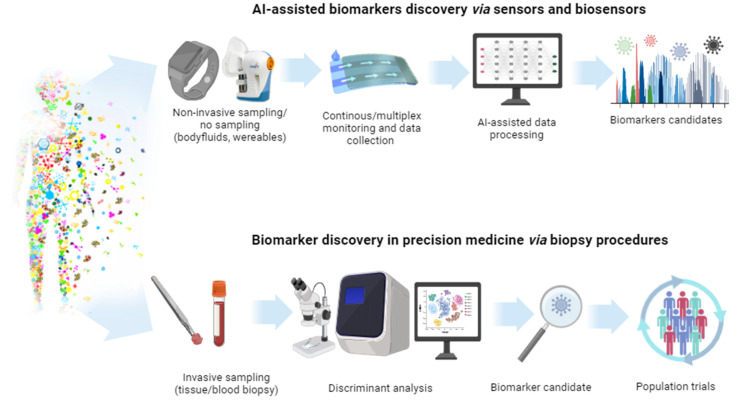
AI-assisted biomarker discovery compared to classic procedures.

To advance biomarker identification, sensors can be designed to monitor specific molecular markers continuously and in real time (Figure 7). To validate the efficacy of this novel sensor, the biofluid’s analysis should be conducted both directly on the subject and verified through standard laboratory methods to approve consistent findings. The sensor must be capable of detecting the relevant physiological concentration ranges, requiring a low detection threshold, high sensitivity, and a broad linear response range. The sensor should be biocompatible and able to discern the target molecule within the complex biofluid environment without the need for preprocessing the sample. This precision is achieved through the use of fixed receptors such as enzymes, peptides, aptamers, or molecularly imprinted polymers (MIPs), which bind specifically to the intended targets [219]. For successful biomarker discovery, it is essential to efficiently gather molecular data from participants. Key obstacles for sensors in accruing accurate data encompass signal interference from physical activity, and the impact of pH, temperature, conductivity, and fluid renewal on sensor performance. Implementing appropriate filtering and calibration methods is essential, as is choosing the right system for fluid sampling. Conducting both longitudinal and cross-sectional studies is critical for biomarker validation, generating extensive datasets. Leveraging contemporary data analysis techniques, such as machine learning, can aid in identifying biomarkers and developing algorithms that predict, diagnose, treat, and prevent diseases.

## 7. Perspectives

Sensors and biosensors as potential PoC devices enable bedside tests, bypassing the need for clinical doctors. These instruments should overcome limitations of dedicated laboratories, catering to timely testing needs in various scenarios. The spread of PoC tests has led to an increase in the number of diagnostic instruments and testing data. However, traditional data management methods remain manual, hampering staff efficiency. An urgent need exists for a standardized data management platform for disease detection. Automated interpretation of abnormal results is essential, especially given the limitations of manual review. In the present era, ML, as a subset of AI, has made remarkable advances. Special emphasis is placed on DL methodologies, such as convolutional neural networks (CNNs) and recurrent neural networks (RNNs). ML offers a solution for classification problems, aiding in interpreting physiological signals and data fusion techniques [154]. AI will play an even more crucial role in handling large multivariate datasets and extracting diagnostic information while avoiding dimensionality issues. ML has been implemented to enhance the specificity of biosensors, effectively substituting the bioreceptor with modeling [220]. Specifically, ML techniques have been implemented to design EN and tongues, and electrochemical, wearable electronics, surface-enhanced Raman spectroscopy (SERS), fluorescence, and colorimetric biosensors [221]. Notably, PCA combined with SVM and various ANN algorithms have demonstrated remarkable performance across diverse tasks. We foresee a steady enhancement of biosensor range through ML and AI, particularly with the potential for sharing trained models and leveraging cloud computing for mobile computation. To support this evolution, greater contributions to open-access data repositories for biosensor data from the biosensing community would be advantageous. A strong link between ML and biosensors should significantly enhance chemometrics for detection, analysis, and diagnosis.

Despite a growing number of applications of AI-based technologies, just a few of them are implemented in the PoC mode today. A bottleneck is mainly the necessity of device validation, data exchange, their confidentiality, and implementation-related logistics [222]. Such applications as i-PROGNOSIS, based on smartphones, are examples of the CovidSense projects, which deal with the analysis of different marker types collected mainly with a smartphone. They respect privacy and data safety policy and provide real benefits for a wide group of patients. The smartphone-based sensors allow the detection of diseases, specified in the European project iPROPELIS (no. 101095697). Using an application, it is possible to identify structural changes in nail plates that indicate psoriasis or psoriatic arthritis (https://cordis.europa.eu/project/id/101095697 accessed on 8 May 2024). Intelligent devices and wearables can be used for the elaboration of new digital biomarkers to create, in addition to already existing solutions, a system for objective monitoring of markers, risk assessment, prognosis, diagnosis, or progress of a disease. It is predicted that in the future, effective AI models will require innovative human–AI/device interfaces capable of collecting and processing the markers in real time, providing the information about the patient’s condition. However, there is an urgent need for simultaneous evaluation of the traditional and digital biomarkers in real time using AI to attain the goals of personalized medicine. A combination of these strategies lies in the utilization of sensors, which can monitor the digital biomarkers (blood pressure, pulse) and the level of traditional biomarkers (glucose, lactate, caffeine, and others). Ultrasonic transducers monitor digital biomarkers and electrochemical sensors measuring the levels of traditional biomarkers. The designed wearable sensor on the skin [223] consisted of both rigid and soft elements, namely non-standard piezoelectric ultrasonic transducers made of lead zirconate, ultrasonic transducers from lead titanate, and printed polymer composites with high mechanical and corrosion resistance. This device is the first step towards multimodal wearable sensors being a combination of acoustic and electrochemical sensors for more complex monitoring of human health. The authors suggest that future development of the self-contained interface integrated with AI/ML would completely convert the current device into an intelligent system wearable on the skin.

Progress in bioelectronics, wearable devices, consumer devices, and digitalization enabled the monitoring of health data in real time and outside conventional clinical conditions. Increasing trust in smart devices propels the demand for these technologies among consumers, researchers, and service providers. The possibility of continuous medical data collection from the natural environment of a patient, which in the past used to be confined to clinical conditions, is supposed to revolutionize and decentralize healthcare. However, continuous monitoring can lead to an overestimation of the health condition of the patient, evoking panic situations. Moreover, variability of sensor output data, namely signal-to-noise ratio and the resulting accuracy, especially in the case of data on a single analyte, can lead to false results because many factors can affect the concentration of a particular biomarker. Since many analytes can be associated with a particular physiological state, multiplex analysis with the integration of digital biomarkers and raw data should generate a more holistic view of the patient’s condition. This kind of complete identification generates very relevant clinical data, which need significant AI/ML intervention regarding sorting and prediction of the results. Accordingly, it is necessary to integrate various technologies, which would support the collection of a broad data spectrum with simultaneous maximization of information accuracy. The key option should be the combination of traditional data with digital biomarkers and their integration with well-trained AI using multiomic data. This should provide a kind of cross-evaluation and combination of personalized biomarkers, which are crucial for a clear evaluation of health condition and minimization of undesired fear-generating factors. That is why there is an urgent but often neglected need for validation, a combination of traditional biomarkers with digital ones, and vice versa. The technologies integrated with AI, with the possibility of multiplexing, would certainly be more consistent with a regulatory mechanism to easily pass through clinical, commercial, and consumer bottlenecks. With all respect for the current research and progress in biosensors and AI/ML, one should ask a question about the target level of clinical and analytical accuracy, which would deem these devices to be good enough to be implemented in clinical practice. The future of biosensors is promising and continuous technological progress results in more accurate and cheaper sensors. For example, the possibility of monitoring glucose level in blood in real time by patients has already changed the practices for healing diabetics.

Finally, some attention should be paid to AI-based language models, which have been the subject of many debates and controversies in recent years. It turns out that it can easily facilitate the correct interpretation of laboratory results. AI-based language models could make diagnoses and plan treatment, but the mistakes made during the calculation of in-vain infusion rate disqualified it as a legitimate ‘online doctor’. Trustworthiness is fundamental in medicine, and as the patient–physician relationship expands into a broader healthcare ecosystem, the introduction of AI prompts a re-evaluation of trust. The creation of trustworthy AI ecosystems is essential. Subtle mistakes can result in serious consequences. On the other hand, ‘real doctors’ can be also mistaken, but in their case, there are legal regulations to force a doctor to care about the patient and to take all necessary precautions. Similarly to mobile health applications, one must differentiate between the application of the language models to high- and low-risk cases, for instance, when diagnosis has a direct impact on patients’ health and life, the supervision and presence of qualified specialists are necessary.

## 8. Conclusions

The field of biomarker detection and their discovery using sensors is notably a multidisciplinary area, requiring a close collaboration among chemists, biologists, engineers, and medical professionals to create advanced, integrated, and multiplexed devices capable of forecasting and averting health issues. We anticipate that advancements in sensor technology will facilitate ongoing, real-time analysis of metabolomics, proteomics, genomics, and other omics fields. The vast amounts of data generated from extensive human studies, combined with effective data fusion and mining techniques, are expected to enhance early disease prediction, diagnosis, and prompt intervention strategies.

Addressing challenges such as data availability and quality, sample size, label variability, privacy concerns, and ethical considerations is essential to fully harness AI’s potential in healthcare. Establishing robust data-driven healthcare systems begins with capturing clean, accurate, and properly formatted data suitable for diverse healthcare applications. There is a widespread belief that large datasets are necessary for accurate predictions, highlighting the importance of high-quality data, thorough annotations, and collaboration with healthcare experts to build reliable ML models. Data security remains a top priority, with healthcare organizations vulnerable to risks such as data breaches, hacking, and ransomware incidents. ML can strengthen data and system security by analyzing patterns to prevent attacks and adapt to evolving threats. Another significant obstacle is the lack of transparency in algorithms and the complexities associated with validation and testing procedures. AI-based applications often show variations from data input to output, and there is currently no standardized procedure in place. Algorithms with similar performance levels may employ different approaches to tackle identical problems, requiring specific preprocessing techniques prior to inference. This diversity complicates scalability, especially in commercial AI-based products, where each application may require its own server or virtual environment. Moreover, ensuring the algorithm’s applicability across different nations faces challenges due to stringent medical regulations.

While many devices concentrate on a single parameter, endeavors should be directed towards simultaneous and noninvasive monitoring of a broad spectrum of biomarkers. This comprehensive analysis not only enables a more thorough examination of physiological states but also facilitates dynamic calibration and correction of responses for more precise monitoring. Biosensors employing multiple sensing methods for the same analyte can also enhance reliability. Digital markers can be used to find patterns and predict medical results thanks to the development of AI. Future progress suggests that digital health platforms will increasingly integrate biosensors into their systems. The patients will be able to share their data with healthcare personnel and monitor their condition in real time, leading to more individualized and prophylactic healthcare. This digital clinical pathway should emphasize the necessity of close interdisciplinary cooperation, where AI can play an important role in integrating different scientific worlds.

## Figures and Tables

**Table 1:** Examples of sensing devices combined with ML algorithms in healthcare applications.

Sensing Device	Type of ML Algorithm	Application	Ref.
Metal oxide-based gas sensor array	RF, K-Nearest Neighbor (KNN), DT, Linear regression, Logistic Regression, Naïve Bayes, LDA, ANN, SVM	Detection, classification, and prediction of concentrations of the four gases simultaneously for disease diagnosis and treatment monitoring.	[168]
Accelerometer sensor embedded in a smartphone	Several ML classifiers	Medical diagnostic, monitoring of users’ daily routine, and detection of abnormal cases	[169]
Accelerometer in wristband	RF	Sleep monitoring	[170]
Zephyr BioHarness for Electrocardiography (ECG)	Batch normalization, SVM, KNN	Cognitive training and stress detection	[171]
ECG, galvanic skin response (GSR), body temperature, SpO2, glucose level, and blood pressure	Neural network model	Psychosocial stress detection	[172]
Optical biosensor	DL	Cancer cell detection	[173]
Electrodermal activity (EDA) and Photoplethysmogram (PPG)	LDA, quadratic discriminant analysis, logistic regression, SVM, Gaussian kernel, KNN, DTs	Hydration monitoring	[174]
Skin temperature, respiratory rate, blood pressure, pulse rate, blood oxygen saturation, and daily activities	Multiple ML techniques	Early detection of COVID-19	[175]
ECG, PPG, and blood pressure (BP)	ResNet with Long short-term memory for hypertension detection	Blood pressure measurement	[176]
Heart rate, heart rate variability, respiration rate, oxygen saturation, blood pulse wave, skin temperature sensors	Multivariate regression for case deterioration	COVID-19 detection	[177]
Inertial measurement unit (IMU) sensor module and plantar pressure	K-means clustering, ANN, SVM	Rehabilitation	[178]
PPG sensor in a ring-type device	DL	Arrhythmia detection	[179]
Plasmonics sensors	Logistic regression, SVM, ANN, CNN, KMM	Cancer detection	[180]
Au nanoparticle-based sensor	SVM	Chronic kidney disease detection	[181]
Electronic nose	ANN	Differentiating lung cancer patients	[182]
Accelerometer and electrodermal activity	SVM	Seizure detection	[183]

## Data Availability

Not applicable.
